# Die Versorgungslandschaft der Kinder- und Jugendpsychiatrie und -psychotherapie in Deutschland: Strukturen, Herausforderungen und Entwicklungen

**DOI:** 10.1007/s00103-023-03724-1

**Published:** 2023-06-13

**Authors:** Renate Schepker, Michael Kölch

**Affiliations:** 1 Abteilung Psychiatrie und Psychotherapie des Kindes- und Jugendalters, Zentrum für Psychiatrie Südwürttemberg, Weingartshofer Str. 2, 88214 Ravensburg, Deutschland; 2 Abteilung Psychiatrie und Psychotherapie des Kindes- und Jugendalters, Universität Ulm, Ulm, Deutschland; 3 Klinik für Psychiatrie, Neurologie, Psychosomatik und Psychotherapie im Kindes- und Jugendalter, Universitätsmedizin Rostock, Rostock, Deutschland

**Keywords:** Kinder- und jugendpsychiatrische Praxen und Institutsambulanzen, Stationäre und teilstationäre Behandlung, Stationsäquivalente Behandlung, Vernetzung und jugendpsychiatrische Verbünde, Child and adolescent private practices and outpatient departments, Inpatient and day patient services, Inpatient equivalent treatment, Networking and associations in Germany

## Abstract

Für die psychiatrische und psychotherapeutische Versorgung von Kindern und Jugendlichen gibt es in Deutschland ambulante, teilstationäre und stationäre Einrichtungen. Eine neue Entwicklung ist die stationsäquivalente Behandlung (StäB), bei der die Patientinnen und Patienten durch ein multiprofessionelles Team in ihrem sozialen Umfeld versorgt werden. In diesem Beitrag wird ein Überblick über die Versorgungslandschaft für psychisch erkrankte Kinder und Jugendliche gegeben. Dabei wird auf geschichtliche Hintergründe sowie auf strukturelle, versorgungspolitische und ökonomische Zusammenhänge eingegangen.

Bis zum Jahr 2014 herrschte im ambulanten Sektor Niederlassungsfreiheit, was dazu führte, dass ländliche Gegenden und sozioökonomisch benachteiligte Stadtteile bis heute teilweise unterversorgt sind. Im Krankenhaussektor nahm zwischen 1991 und 2004 die Bettenzahl stark ab und danach zugunsten besserer Regionalisierung mit kleinen Einheiten und kleinen Stationen wieder deutlich zu, ergänzt durch überproportional gewachsene Tagesklinikplätze. Stationsäquivalente Behandlungen sind ebenso effektiv, aber noch nicht flächendeckend etabliert, Modellvorhaben nur wenige verhandelt. Regionale Vernetzungen aller Hilfesysteme bis hin zu anzustrebenden jugendpsychiatrischen Verbünden erfahren in der Versäulung des Sozialsystems liegende Begrenzungen. Als Fazit wären ein Kooperationsgebot über die Sozialgesetzbücher hinweg und die Ermöglichung wahrer sektorenübergreifender Versorgung wünschenswert.

## Abstract (en)

For children and adolescents in need of psychiatric and psychotherapeutic care, outpatient, day patient, and inpatient facilities are provided. A new development is called “inpatient equivalent treatment” that consists of home visits by a multiprofessional team. This paper depicts the landscape of Child and Adolescent Psychiatry (CAP) Services, covering its historical development and the structural, care policy, and financing backgrounds. Until 2014, there was free choice of private practice locations in the outpatient sector, leaving rural areas and marginalized neighborhoods partially undersupplied until today.

The number of beds in the hospital sector decreased significantly between 1991 and 2004. It later rose again in favor of improved regional access and smaller units, with an additional 50% of day patient places. Inpatient equivalent treatments are equally effective, but not yet established nationwide; only a few innovative models have been negotiated. Regional networks of all social support systems, aiming for child psychiatric supply networks, are limited due to the pillarization of the social system. In conclusion, an imperative cooperation between all services of the Social Security Code and enabling true cross-sectoral services would benefit CAP patients.

## Einleitung

Für das Fachgebiet der Kinder- und Jugendpsychiatrie und -psychotherapie (KJPP) gelten besondere versorgungstechnische Voraussetzungen, an denen die Güte der Versorgung überprüft werden kann. Es muss berücksichtigt werden, dass viele der zu behandelnden Kinder und Jugendlichen aus sozioökonomisch benachteiligten Regionen stammen. Wohnortnahe Angebote sind wichtig, da die Familien in die Behandlung einbezogen werden sollen und die Einrichtungen gut erreichbar sein müssen. Auch muss Sorge für multiple Vernetzungen mit anderen Helfersystemen getragen werden. Nicht zuletzt müssen durch die Qualität der Versorgung schnell drohende Chronifizierungen von Störungen verhindert werden können.

Im vorliegenden Beitrag sollen die verschiedenen Versorgungsmöglichkeiten und -strukturen der Kinder- und Jugendpsychiatrie und -psychotherapie in Deutschland dargestellt werden. Dabei wird auf geschichtliche, strukturelle, versorgungspolitische und ökonomische Zusammenhänge sowie auf die sich daraus ergebenden Defizite eingegangen.

## Ambulante Versorgung

Da sich das Fachgebiet der Kinder- und Jugendpsychiatrie vergleichsweise spät herausgebildet hat und die Gebietsbezeichnung erst seit 1968 existiert, hat sich die ambulante Versorgung durch niedergelassene Kolleginnen und Kollegen erst seit den 1980er-Jahren entwickelt [1]. Es herrschte infolge der geringen Zahl von weniger als 1000 Niedergelassenen bis 2014 Niederlassungsfreiheit. Das hat dazu geführt, dass eine ungleiche Verteilung der Kassensitze mit Bevorzugung der großen oder attraktiven Städte entstanden ist. Neben ländlichen Gebieten sind innerhalb der Städte sehr oft Problemstadtteile unterversorgt (z. B. in Berlin, München), so dass das „inverse care law“ (Gesetz der umgekehrten Versorgung) im Fachgebiet immer wieder thematisiert wird: Viele Ressourcen befinden sich eben nicht dort, wo die Kinder wohnen, die ihrer am meisten bedürfen. Ebenfalls ist nach wie vor eine Unterversorgung der östlichen relativ zu den westlichen Bundesländern hinsichtlich der ambulanten Kapazitäten zu verzeichnen. Berg [1] wendet allerdings ein, dass solche einfachen Aussagen bei genauerer Betrachtung nicht vollumfänglich zutreffen. Gleichwohl errechnet er, dass der „Versorgungsquotient“ (Behandlungsfälle pro Gesamtzahl der Einwohnenden bis 18 Jahre × 1000) zwischen den Bundesländern Sachsen und Berlin um den Faktor 5 differiert, zwischen Sachsen und Hamburg sogar um den Faktor 6,7.

Kinder- und Jugendpsychiaterinnen und -psychiater zählen in der kassenärztlichen Versorgung zur „spezialisierten fachärztlichen Versorgung“, d. h., sie werden nicht auf Kreisebene, sondern in 97 „Raumordnungsregionen“ in Deutschland geplant. Zielgröße ist seit dem 19.08.2022 eine Verhältniszahl von 15.210 Einwohnenden unter 18 Jahren je Ärztin oder Arzt. Die Verhältniszahl wurde am 21.04.2022 vom Gemeinsamen Bundesausschuss zur Verbesserung der Versorgungssituation und zum Ausgleich der „relativ heterogene[n] Verteilung der Kinder- und Jugendpsychiater, die bereits vor Einführung der Bedarfsplanung bestand und bis heute aufgrund der geringen Arztzahl noch immer besteht“, um 10 % verbessert [2]. Die Entscheidung wird folgendermaßen begründet: „Die Rückmeldungen aus der kinder- und jugendpsychiatrischen Versorgung machen deutlich, dass das Versorgungsniveau zum Zeitpunkt der Aufnahme der Kinder- und Jugendpsychiater in die Bedarfsplanung nicht mehr ausreicht, um den Versorgungsbedarf zu decken. Immer mehr Kinder und Jugendliche haben Schwierigkeiten, zeitnah Termine beim Kinder- und Jugendpsychiater zu erhalten. Im Gutachten zur Weiterentwicklung der Bedarfsplanung vom 20.09.2018 wurde festgestellt, dass die potenziellen Wegezeiten zu einem Kinder- und Jugendpsychiater im Arztgruppenvergleich sehr hoch liegen. Kinder- und Jugendpsychiater sind von etwa 4 % der Bevölkerung (bezogen auf Bevölkerung unter 18 Jahren) erst in minimal 45 min erreichbar.“ Hierzu wird auf Sundmacher et al. [3] verwiesen. Diese Versorgungssituation hatte dazu geführt, dass zumindest bis 2015 die „Behandlungsquoten“ für psychische Störungen bei Kindern und Jugendlichen insgesamt niedriger lagen als bei Erwachsenen [4].

Derzeit sind bundesweit in der Kinder- und Jugendpsychiatrie etliche „Facharztsitze“ unbesetzt. Tätig sind laut der Ärztestatistik der Bundesärztekammer 1062 niedergelassene Fachärztinnen und -ärzte für Kinder- und Jugendpsychiatrie und -psychotherapie, entsprechend 0,93 % der gesamten niedergelassenen Ärzteschaft. Kinder- und Jugendpsychiaterinnen und -psychiater arbeiteten noch im Jahr 2021 nur zu 26 % in Berufsausübungsgemeinschaften, d. h. in größeren Praxen mit mehreren Fachärztinnen und Fachärzten (verglichen mit 40 % aller Kinderärztinnen und -ärzte). Die meisten Kinder- und Jugendpsychiaterinnen und -psychiater hingegen arbeiten in sozialpsychiatrischen Praxen gemäß der Sozialpsychiatrie-Vereinbarung^1^ des Fünften Buchs Sozialgesetzbuch (SGB V). Diese erlaubt es einer Fachärztin oder einem Facharzt, mindestens 1,5 nichtärztliche, pädagogisch-therapeutisch tätige Mitarbeitende einzustellen, welche dann im Sinne des Gesamttherapieplans supervidiert tätig werden. Dadurch arbeiten inzwischen in der Mehrzahl der Praxen Teams von bis zu 15 Mitarbeitenden. Dies ermöglicht eine multiprofessionelle Behandlung entsprechend den individuellen Bedürfnissen der Patientinnen und Patienten. Die Leistungen der nichtärztlichen Mitarbeitenden werden pauschal vergütet. Die Anzahl der Patientinnen und Patienten, die sozialpsychiatrisch behandelt werden können, ist mit der Vereinbarung auf 150 im Quartal gedeckelt. Die Vereinbarung können nicht nur Kinder- und Jugendpsychiaterinnen und -psychiater, sondern auch Kinderärztinnen und -ärzte oder Erwachsenenpsychiaterinnen und -psychiater mit mindestens 2‑jähriger Erfahrung in Kinder- und Jugendpsychiatrie und -psychotherapie abschließen.

Institutsambulanzen an Kliniken nehmen in sehr unterschiedlichem Umfang an der ambulanten Versorgung teil und unterliegen bundesweit sehr differenten Finanzierungsregelungen, was die Leistungsmengen fördern oder begrenzen kann. Die Einschlusskriterien für die Behandlung von Kindern und Jugendlichen sind aufgrund der allgemein geringen Versorgungsdichte weniger restriktiv gewählt als für Erwachsene. Auch wenn es um die Verkürzung oder Vermeidung eines stationären Aufenthalts oder die Sicherung der therapeutischen Kontinuität geht, sind Behandlungen in Institutsambulanzen angezeigt. Derzeit werden die Leistungen in den meisten Bundesländern pauschal vergütet, wobei die Vergütungshöhen recht unterschiedlich ausfallen. Eine Vereinheitlichung zeichnet sich bisher nicht ab.

Die Altersgrenzen der Patientinnen und Patienten differieren je nach Subsystem der Versorgung: Niedergelassene Kinder- und Jugendpsychiaterinnen und -psychiater können Kinder und Jugendliche bis zum 21. Geburtstag behandeln, Institutsambulanzen nur bis zum 18. Geburtstag. Bei zuvor schon bestehenden längeren Behandlungen können im Einzelfall noch 1–2 Quartale bis zum Abschluss oder zur Überleitung in Erwachsenenkontexte gewährt werden.

Zu anderen, verwandten Leistungserbringern der ambulanten Versorgung bestehen in aller Regel enge regionale Kooperationsbeziehungen, wie zu Kinder- und Jugendlichenpsychotherapeutinnen und -therapeuten, sozialpädiatrischen Zentren, Kinderärztinnen und -ärzten mit fachgebundener Psychotherapie oder Beratungsstellen in verschiedener Trägerschaft. Diese sind – mit regionalen Ausnahmen – im Bundesdurchschnitt weitaus zahlreicher als die Ärztinnen und Ärzte für Kinder- und Jugendpsychiatrie und -psychotherapie. So kommen z. B. im Durchschnitt etwa 5 Kinder- und Jugendlichenpsychotherapeutinnen und -psychotherapeuten auf 1 Kinder- und Jugendpsychiaterin/-psychiater.

In komplexen Einzelfällen sind derzeit bestehende „Leistungsausschlüsse“ hinderlich. So kann eine Patientin oder ein Patient nicht gleichzeitig eine Psychotherapie bei einer/m Kinder- und Jugendlichenpsychotherapeutin/-therapeuten und eine Behandlung in einer Institutsambulanz wahrnehmen. Ebenso kann nicht gleichzeitig in einer Institutsambulanz und in einer sozialpsychiatrischen Praxis behandelt werden – obwohl dies, etwa für spezialisierte Gruppenangebote oder auch zum Monitoring einer besonders komplexen Medikation, in Einzelfällen sehr sinnvoll sein kann. Institutsambulanzen können gleichwohl Niedergelassene stundenweise einstellen und dadurch rückvergüten. Ähnliche Leistungsausschlüsse existieren für sozialpädiatrische Zentren. Entstehende Kooperationsbeziehungen gehen im Einzelfall darüber hinaus und betreffen Mitarbeitende in Jugendhilfeeinrichtungen, Lehrerinnen und Lehrer sowie Schulsozialarbeiterinnen und -sozialarbeiter und auch Schul- und Jugendämter oder Arbeitsagenturen (s. unten). Sie werden sämtlich auf freiwilliger Basis getroffen, denn anders als im Elften Buch Sozialgesetzbuch (SGB IX) besteht im SGB V kein „Kooperationsgebot“.

Es besteht seitens der Fachverbände die Hoffnung, dass die Hindernisse für die Versorgung v. a. schwer psychisch erkrankter Kinder und Jugendlicher im Rahmen der noch im Jahr 2023 für Kinder und Jugendliche zu erarbeitenden ambulanten berufsgruppenübergreifenden Komplexbehandlungsrichtlinie gemäß § 92 Absatz 6b SGB V verringert werden können.

## Krankenhäuser: Stationäre, teilstationäre und stationsäquivalente Behandlung

Die Versorgung psychisch kranker Kinder und Jugendlicher findet überwiegend im ambulanten Sektor statt, was meist auch sinnvoll ist, weil stationäre Behandlungen den Nachteil einer Trennung aus der familiären und weiteren Lebenswelt bedeuten. Gelegentlich ist aber eine stationäre Versorgung aus diagnostischen oder therapeutischen Gründen angezeigt.

Gegenläufig zur ambulanten Versorgung hat die vollstationäre Versorgung von 1991 bis 1994 nach der Einführung der Psychiatrie-Personalverordnung (Psych-PV) zunächst einen massiven Bettenabbau erlebt (−41,9 %). Von 1995 bis 2004 kam nur noch ein weiterer Bettenabbau von −0,5 % hinzu. Die enorme Abnahme lässt sich überwiegend auf den Abbau von Langzeitplätzen, die Intensivierung von Therapieprozessen und die Verkürzung der durchschnittlichen Verweildauer (um −65,5 %, dann nochmals um −31,2 %) zurückführen. Dabei wurde in den Pflegesatzverhandlungen die gleichzeitig erhöhte Fallzahl (+82,9 % sowie im 2. Zeitraum nochmals +57,8 %) nach den Umfrageergebnissen der Aktion Psychisch Kranke [5] nicht abgebildet, denn die vereinbarten Fallzahlen blieben hinter den realen zurück.

Seit 2004 besteht nun ein kontinuierlicher Bettenzuwachs bei weiterhin sinkender Verweildauer und sich erhöhenden Fallzahlen im Fachgebiet der Kinder- und Jugendpsychiatrie. 2021 lag laut Statistischem Bundesamt^2^ die Bettenzahl bei 6702 (+39 % gegenüber 2004), die durchschnittliche Verweildauer bei 32,9 Tagen (−25 % gegenüber 2004) und die Fallzahl bei 63.820 (+76 % gegenüber 2004; [6]). Der Bettenzuwachs war zunächst der Sicherstellung einer flächendeckenden Versorgung und Erreichbarkeit geschuldet, aber auch zunehmenden Notfallquoten.

Die Notfallaufnahmen sind während der Coronapandemie bundesweit nicht gestiegen, sie beliefen sich – definiert nach dem im Abrechnungsdatenfile angegebenen Aufnahmegrund „N“ – im 1. Halbjahr 2021 auf 48,9 % aller Fälle [7]. Dem widerspricht nicht, dass regional deutliche Engpässe mit stark erhöhtem Aufnahmedruck entstanden sind – vor allem in Regionen mit sehr niedriger Bettenmessziffer wie in Stadtstaaten oder in Süddeutschland [8, 9]. Die Auslastung der Kapazitäten ist in Deutschland unverändert mit 85,6 % [6] sehr hoch und ging pandemiebedingt – im Gegensatz zu allen anderen Krankenhausabteilungen – zwischen 2019 und 2021 nur leicht zurück. Zum Vergleich lag die Auslastung in der Pädiatrie 2021 bei 55,2 % [6]. In den Vorjahren lag sie in der Kinder- und Jugendpsychiatrie regelhaft bei über 90 %.

Stationäre Kapazitäten werden überwiegend mit kleinen Stationseinheiten (10–14 Betten je Station) und altersgruppenbezogen geführt; dabei ist eine Station in der Kinder- und Jugendpsychiatrie zwar deutlich kleiner als vergleichsweise in der Erwachsenenpsychiatrie (die doppelte Stationsgrößen führt), aber größer als die regelhaft nicht mehr als 8 Plätze umfassenden Wohngruppen in Jugendhilfeeinrichtungen. Jugendpsychiatrische Kliniken oder Abteilungen mit mehr als 60 Betten sind heute wesentlich seltener in der Versorgungslandschaft vertreten als kleinere Einheiten. Die durchschnittliche Größe liegt um die 40 Betten, der Median noch darunter.

Spezialisierungen sind innerhalb der Versorgungslandschaft rar. Spezialkliniken und -stationen für intelligenzgeminderte Kinder und Jugendliche existieren in einigen Bundesländern mit Sonderversorgung (z. B. Baden-Württemberg, Bayern, Schleswig-Holstein, Sachsen-Anhalt). Sie wurden z. B. in Nordrhein-Westfalen im Zuge der Umsetzung der UN-Behindertenkonvention abgeschafft. Sie haben dennoch für die jeweiligen Patientinnen und Patienten den Vorteil eines spezifischen Milieus mit eigener „Taktung“ und Umgebung und können darin hocheffektive Arbeit leisten.

Jugend-Sucht-Stationen mit einem besonderen milieutherapeutischen Setting wurden ab den 1970er-Jahren gegründet und existieren derzeit an mehr als 20 Standorten. Sie nehmen keine Notfallzuweisungen auf – jugendliche Patientinnen und Patienten mit akuten Intoxikationen werden wegen der unklaren Dosis-Wirkungs-Beziehungen und häufigen Mischintoxikationen eher in Kinderkliniken, internistischen Kliniken oder je nach regionalen Absprachen auch in der jugendpsychiatrischen Regelversorgung behandelt. Bundesweit existieren Standards zum klinischen Vorgehen bei Jugendlichen mit Substanzstörungen [10] und im Operationen- und Prozedurenschlüssel (OPS; [11]) hat diese Behandlungsform einen eigenen OPS-Code (9-694) erhalten, da sie aufwändiger ist als andere Behandlungen und besondere Qualitätsmerkmale erfüllen muss. Insbesondere sind – wegen der regelhaften Komorbiditäten neben den Suchtproblemen – eine fachärztliche Leitung und spezifische gruppentherapeutische sowie fachtherapeutische Angebote erforderlich.

In der Regel haben alle Abteilungen bzw. Kliniken für Kinder- und Jugendpsychiatrie und Psychotherapie die regionale Pflichtversorgung für ein definiertes Einzugsgebiet inne, bis auf 2 Ausnahmen (München, Berlin) auch die Universitätskliniken. Das bedeutet eine Verpflichtung zur Notfallbehandlung, zur Aufnahme von Jugendlichen, die unter geschlossenen Bedingungen geführt werden müssen, und zur Regelbehandlung der krankenhausbedürftigen Kinder und Jugendlichen der Region. Dabei werden die meisten Stationen als „fakultativ geschlossen“ geführt und ein nahtloser Übergang von „geschützter“ zu offener Behandlung der betroffenen Patientinnen und Patienten ist jederzeit möglich. Die familienrechtlichen Rahmenbedingungen, die für Kinder und Jugendliche unabhängig von der Rechtsgrundlage einer Unterbringung gelten, erlauben hier eine höhere Flexibilität als bei Erwachsenen.

Die Versorgungsdichte ist im vollstationären Sektor ebenso heterogen wie im ambulanten. Bettenzahlen pro minderjährigen Einwohnenden schwanken zwischen den Bundesländern um den Faktor 3 [12]. Die Verhältniszahlen lagen 2019 zwischen 3,49 vollstationären Betten pro 10.000 Einwohnenden unter 18 Jahren (Bayern) und 10,64 Betten pro 10.000 (Sachsen-Anhalt). Hinzu kommen, ebenso wie im ambulanten Sektor, erhebliche regionale Unterschiede auch innerhalb der Bundesländer. Deutschland hält dabei mit durchschnittlich 4,9 Betten pro 10.000 Einwohnenden unter 18 Jahren europaweit eine Spitzenposition. England ist mit nur 0,94 Betten/10.000 und Schweden mit nur 0,12 Betten demgegenüber deutlich sparsamer aufgestellt [13].

Das vollstationäre Angebot wird seit den 1980er-Jahren ergänzt durch zunehmende teilstationäre Einheiten, die oft zur Erschließung der großen Versorgungsgebiete dezentral mit Entfernung von der Stammklinik geführt werden und in der Regel eine eigene Institutsambulanz führen. Dabei kann das Verhältnis teilstationärer zu vollstationärer Kapazität durchaus 1:2 bis nahe an 1:1 betragen, was ungefähr der aktuellen Spannbreite im Bundesgebiet entspricht.

Tageskliniken bieten sich vor allem für Kinder an, die den täglichen Milieuwechsel bewältigen können und deren Familien stabil genug sind, mit der Symptomatik der Kinder weiter umzugehen. Das Behandlungsangebot ist in der Regel tagsüber ebenso intensiv wie im vollstationären Bereich und pausiert an den Wochenenden. Teilstationäre Angebote müssen für die Patientinnen und Patienten und deren Eltern in einem realistischen Zeitraum erreichbar sein – die Finanzierung der Fahrten ist eine Kann-Leistung der Kostenträger.

Auch hier hat sich ein deutlicher Aufwuchs an teilstationärer Kapazität entwickelt, von 1454 Plätzen im Jahr 2004 bis auf 3652 Plätze 2017, was einer Zunahme von 151 % entspricht. Teils sind diese Plätze durch „integrierte“ teilstationäre Behandlungen auf Regelstationen geschaffen worden, was wiederum der Behandlungskontinuität in der Entlassungsvorbereitung dienen kann.

Der Ausbau an tagesklinischen Plätzen führte allerdings nicht im erwarteten Ausmaß zur Entlastung vollstationärer Kapazitäten, stattdessen scheinen durch die teilstationären Plätze insgesamt mehr Kinder und Jugendliche mit psychischen Auffälligkeiten erreicht zu werden [14], was vermuten lässt, dass Angebote in größerer räumlicher Nähe besser angenommen werden.

Familien sollten regelhaft in jede Behandlung einbezogen werden. An einigen Kliniken sind im Rahmen eines besonderen Angebots Eltern-Kind-Stationen oder Eltern-Kind-Tageskliniken etabliert, auf denen Eltern(teile) mitaufgenommen werden können. Diese Behandlungsform ist bei Familien indiziert, in denen die Eltern-Kind-Dynamik wesentlich zur Entstehung oder Aufrechterhaltung der kindlichen Symptomatik beiträgt. Seit 2021 ist – in Kooperation mit der Erwachsenenpsychiatrie – als besondere Behandlungsform eine bifokale Eltern-Kind-Behandlung zugelassen, bei der ein stationär behandlungsbedürftiger Elternteil und dessen stationär behandlungsbedürftiges Kind gleichzeitig und gemeinsam von Spezialisten zweier Fachgebiete behandelt werden, unter der Ziffer des Operationen- und Prozedurenschlüssels (OPS) 9‑64a. Solche Spezialangebote existieren noch nicht an vielen Orten, erfreuen sich aber zunehmender Aufmerksamkeit.

Als neueste Entwicklung kinder- und jugendpsychiatrischer Behandlungsleistungen durch die Krankenhäuser wurde die stationsäquivalente Behandlung (StäB) ab 2017 mit dem Gesetz zur Weiterentwicklung der Versorgung und der Vergütung für psychiatrische und psychosomatische Leistungen (PsychVVG) ermöglicht und war ab dem 01.01.2018 finanziell umsetzbar [15]. Sie besteht aus einem mobilen aufsuchenden, fachärztlich geleiteten multiprofessionellen Team, an dem mindestens 3 Berufsgruppen beteiligt sind. Dieses Team leistet an 7 Tagen in der Woche Kontakte zu den – stationär behandlungsbedürftigen – Patientinnen und Patienten in deren (Aufenthalts‑)Familie und im sozialen Umfeld und bietet indikationsspezifisch, multiprofessionell Einzel- und auch Gruppentherapieangebote an. Die Patientinnen und Patienten erhalten wöchentlich ärztliche Visiten, sie können die Schule für Kranke oder weiter ihre Herkunftsschule besuchen. Das Team steht bis abends, die anbietende Krankenhausabteilung jederzeit für Kriseninterventionen bereit.

Laut den Erfahrungen von Boege et al. [15] sind alle Störungsbilder im Rahmen einer StäB behandelbar. Die Diagnosenverteilung war im Jahr der Einführung mit jener der vollstationären Klientel vergleichbar und die Behandlungsform zeichnete sich durch eine hohe Haltequote von 86 % und eine hohe Patienten- und Elternzufriedenheit aus. Die durchschnittliche Behandlungsdauer von 37,9 Tagen fiel bei Boege et al. [15] kürzer aus als eine vollstationäre Regelbehandlung, womit StäB trotz des logistischen und Fahrzeitaufwandes ökonomischer ist. Zur Wirksamkeit von Home-Treatment-Ansätzen, die StäB zugrunde liegen, besteht bereits seit Jahren eine gute Evidenzlage [15]. Eine Reduktion stationärer Betten gelang trotz Einführung der neuen Behandlungsform laut Boege et al. [15] nicht, wobei die untersuchte Region zu den kapazitätsmäßig eher geringer ausgestatteten gehört. Dem StäB-Ansatz würde man mehr Verbreitung wünschen – seitens der gesetzlichen Krankenversicherung ist jedoch im ersten „Gemeinsamen Bericht“ mit der Deutschen Krankenhausgesellschaft an das Bundesgesundheitsministerium – entgegen den Erfahrungen gerade aus der Kinder- und Jugendpsychiatrie – eine negative Bewertung erfolgt [16], die nicht unwidersprochen blieb [17]. Derzeit muss die Finanzierung dieser Leistung von jedem Träger neu verhandelt werden.

Modellvorhaben nach § 64b SGB V, die über die geschilderten traditionellen ambulanten und krankenhausbasierten Leistungen hinaus noch weit flexiblere Ansätze ermöglichen (z. B. intermittierende teilstationäre Behandlungen; aufsuchende Behandlung, wenn erforderlich, aber nicht täglich; Adoleszenzpsychiatrie mit gleitenden Übergängen), haben sich in der Kinder- und Jugendpsychiatrie entgegen dem gesetzlichen Ziel nicht in jedem Bundesland etablieren können. Bisher existieren nach Wissen der Autorin und des Autors nur 8 Modellvorhaben, was einerseits an den dafür notwendigen langwierigen Verhandlungen liegt. Andererseits sind die Vorhaben meist nur mit einzelnen Kassen verhandelt/verhandelbar, was eine regional gleichmäßige Nutzung des Angebots verunmöglicht und eine Umsetzung erschwert, da das Angebot nur für wenige Patientinnen und Patienten nutzbar ist.

Eine Übersicht bisheriger Erfahrungen aus dem Fachgebiet KJPP bieten Schwarz et al. [18], die vor allem die bessere Behandlungskontinuität und die stärkere Einbeziehung und damit höhere Zufriedenheit der Sorgeberechtigten betonen. Zukunftsweisend wäre eine maximale Flexibilität bei der Verwendung von Regionalbudgets aller Kostenträger. Hier konnte erstmals im Jahr 2020 ein großes Regionalbudget in Rheinland-Pfalz im Rahmen des § 64b SGB V verhandelt werden, das die Kinder- und Jugendpsychiatrie inkludiert [19] Dazu können jedoch noch keine Erfahrungen berichtet werden.

Außerhalb der Modellvorhaben und der StäB ist die Finanzierung kinder- und jugendpsychiatrischer Behandlungen über die Bundespflegesatzvereinbarung, die OPS und die PEPP^3^ geregelt. Die Richtlinie Personalbemessung in Psychiatrie und Psychotherapie (PPP-Richtlinie)^4^ existiert und wird aktuell sanktionsfrei überprüft, deren ausfinanzierte Budgetrelevanz ist derzeit jedoch strittig. Strukturvorgaben der OPS-Codes werden regelhaft von den medizinischen Diensten kontrolliert. Bisher mussten angesichts des auch hier steigenden Fachkräftemangels, anders als bereits in der Versorgung durch Kinderkliniken zu bedauern, nur punktuell Kliniken für Kinder- und Jugendpsychiatrie und -psychotherapie schließen. Der Bedarf an Behandlungen ist auch während der Coronapandemie ungebrochen hoch geblieben [7].

## Zusätzliche Maßnahmen: Entwicklungen und Entwicklungsdesiderata

Der Begriff der „komplementären Strukturen“ ist im Fachgebiet der Kinder- und Jugendpsychiatrie nicht gebräuchlich, auch eine „Behandlungskette“: ambulante Versorgung – stationäre Behandlung – teilstationäre Behandlung – Rehabilitation, wäre nicht einfach auf die Bedarfe von Kindern und Jugendlichen zu übertragen oder zu realisieren. Vielmehr ist die Jugendhilfe traditionell als Rehabilitationsträger für psychisch behinderte oder von Behinderung bedrohte Kinder zuständig, entzieht sich aber durch das Achte Buch Sozialgesetzbuch (SGB VIII) als eigenständige Struktur etwa ärztlichen Zuweisungen oder auch nur gemeindepsychiatrischer Hilfeplanung wie im Erwachsenenbereich, da die Jugendhilfeplanung eigenständig kodifiziert ist. Für intelligenzgeminderte Kinder und Jugendliche ist wiederum die Eingliederungshilfe zuständig, teilweise müssen zur Zuständigkeit rechtliche Auseinandersetzungen geführt werden. Große Hoffnungen hinsichtlich einer Vereinheitlichung der Vorgehensweisen liegen nunmehr bei der Umsetzung der „inklusiven Lösung“ nach der Reform des SGB VIII [20].

Auch das Schulsystem ist als wichtiger Realraum für Kinder und Jugendliche auf Länderebene sehr unterschiedlich aufgestellt. Die Gewährleistung des Unterrichts während einer Krankenhausbehandlung ist bis heute nicht in allen Bundesländern gegeben. Ebenso verhält es sich mit wichtigen Kooperationspartnern wie der Schulsozialarbeit oder schulpsychologischen Diensten oder mit jugendpsychiatrischen Diensten der Gesundheitsämter, welche auf regionaler Ebene sehr unterschiedlich ausgebaut oder gar nicht vorhanden sind. Diese sind mit den ambulanten oder krankenhausbasierten Leistungserbringern in sehr unterschiedlicher Weise vernetzt. Das Gleiche gilt für die – in aller Regel überregional arbeitenden – Rehabilitationskliniken für psychisch kranke Kinder und für aktuell entstehende, erste Modelle ambulanter Rehabilitation.

Arbeitsagenturen und Berufsanbahnungseinrichtungen, wie Berufsbildungswerke, sind für Jugendliche oft wesentlich hinsichtlich ihrer Orientierung auf eine Arbeitstätigkeit. Hier sind die Kooperationen in den letzten Jahren deutlich gewachsen, wenngleich im Evaluationsbericht des Behindertengleichstellungsgesetzes bemängelt wird, dass die Kompetenz für den Umgang mit psychisch Kranken nicht in allen Behörden schon gut ausgebildet ist.^5^

Jugendhilfeeinrichtungen haben regelhaft keine „Versorgungsverpflichtung“ und es bedarf zur Versorgung der dortigen „psychiatrischen Hochrisikoklientel“ vielfältiger Kooperationsabsprachen. Schrittweise etablieren sich aufsuchende jugendpsychiatrische Konsiliardienste in Jugendhilfeeinrichtungen.

All die genannten Beteiligten wären regional in „jugendpsychiatrische Verbünde“ verschiedener Hilfeerbringer mit den Kinder- und jugendpsychiatrischen Leistungserbringern aus allen Sektoren zwingend einzubeziehen. Solche Verbünde sind rechtlich nur im Psychisch-Kranken-Hilfegesetz in Baden-Württemberg vorgesehen, harren aber dort ebenfalls der Umsetzung. Die aus der Sektoren- und sozialrechtlichen Versäulung entstehenden multiplen Schnittstellenprobleme, bei denen gelegentlich für behandlungsbedürftige Kinder und Jugendliche „schwarze Löcher“ der Zuständigkeit entstehen können, wurden bereits im 13. Kinder- und Jugendhilfebericht 2009 moniert [21].

## Fazit

Die kinder- und jugendpsychiatrische Versorgung in Deutschland ist im europäischen Vergleich sehr gut ausgebaut, aber noch zu wenig auf die sektorübergreifende und auf die ambulante Versorgung ausgerichtet. Sie hat insgesamt in den letzten Jahrzehnten wesentliche Differenzierungen erfahren. Hinderlich sind Leistungsausschlüsse, Sektorengrenzen und in der Versäulung des Sozialsystems liegende Begrenzungen für eine bessere Nutzung der vorhandenen Ressourcen auch gemeinsam mit den unterstützenden Systemen und Behörden. Vor allem bei den schwerer beeinträchtigten Patientinnen und Patienten mit komplexem Behandlungsbedarf wäre zugunsten eines gemeinsamen Fallverständnisses und einer gemeinsamen Behandlung über Sektorengrenzen, auch über die Grenzen der Sozialgesetzbücher hinweg, eine besser koordinierte Versorgung im Sinne einer gemeinsamen Behandlung zu wünschen.

## Footnotes

1 Vereinbarung gemäß § 85 Abs. 2 Satz 4 und § 43 a SGB V über besondere Maßnahmen zur Verbesserung der sozialpsychiatrischen Versorgung von Kindern und Jugendlichen (Sozialpsychiatrie-Vereinbarung – Anlage 11 zu den Bundesmantelverträgen).

2 Anmerkung: Auch die von der Aktion Psychisch Kranke e. V. (APK) 2007 verwendeten weiter oben genannten Daten [5] entstammten den Angaben des Statistischen Bundesamtes, so dass die Datenbasis vergleichbar ist.

3 Pauschalierendes Entgeltsystem Psychiatrie und Psychotherapie, s. Website des Instituts für das Entgeltsystem im Krankenhaus InEK; https://www.g-drg.de/pepp-entgeltsystem-2022 (zuletzt besucht am 04.01.2023).

4 Herausgegeben und jährlich revidiert vom Gemeinsamen Bundesausschuss (G-BA), https://www.g-ba.de/richtlinien/113/ (zuletzt besucht am 04.01.2023).

5 Siehe eine Stellungnahme der Bundesregierung dazu: https://dserver.bundestag.de/btd/20/044/2004440.pdf (zuletzt besucht am 30.03.2023).
